# More Than Defense in Daily Experience of Privacy: The Functions of Privacy in Digital and Physical Environments

**DOI:** 10.5964/ejop.v12i1.948

**Published:** 2016-02-29

**Authors:** Debora Benedetta Lombardi, Maria Rita Ciceri

**Affiliations:** a Department of Psychology, University of the Sacred Heart of Milan, Milan, Italy; University of Neuchâtel, Neuchâtel, Switzerland

**Keywords:** privacy, positive experience, functions for well-being, person-environment interaction

## Abstract

The purpose of the current study was to investigate the experience of privacy, focusing on its functional role in personal well-being. A sample (N = 180) comprised subjects between 18 and 50 years of age were asked to spontaneously provide accounts of their experiences with privacy and answer close-ended questions to acquire a description of a daily experience of privacy. The results showed the importance attributed to the function of privacy related to the “defense from social threats”, and the twofold function of privacy related to an “achieved state of privacy”, in the terms of both “system maintenance” and “system development”. The results also shed light on the role of the environment in shaping one’s experience of privacy. Specifically, the participants recognized more easily the function of defense from threats related to seeking privacy while interacting in digital environments, whereas they seemed to benefit from positive functions related to an achieved state of privacy in physical environments. The findings sustain the notion of privacy as a supportive condition for some psychological processes involved in the positive human functioning and confirm previous studies conducted on the role of privacy in human well-being.

In complex societies, where the boundaries between physical and digital environments are difficult to distinguish, the definition of privacy and the knowledge of its beneficial role in individuals’ well-being become challenging matters. The current study focused on the role of the experience of privacy in personal well-being in order to explore its functions in both digital and physical environments.

Privacy is a condition strongly related to everyday life. It plays a crucial role in enabling people to manage both their social interactions and their personal activities (Pedersen, 1997).

Despite the lack of a univocal conceptual framework on the definition of privacy, scholars consider privacy a central ingredient in the positive human functioning (Altman, 1975; Newell, 1994). Social and cognitive studies agree that privacy is a voluntary and temporary condition of separation from public domain (Newell, 1994, 1995, 1998) that originates from the interaction between the individual’s features (e.g., need for recovery, need for self-regulation; Johnson, 1974; Pedersen, 1999) and the environment (e.g., Is this place supportive for the satisfaction of my need for recovery?) (Cole & Hall, 2010; Demirbas & Demirkan, 2000; Korpela, Kyttä, & Hartig, 2002; Palen & Dourish, 2003).

Accordingly, we consider privacy a socio-environmental construct, as the person-environment interaction determines both the shape of privacy, (i.e., thus, if privacy takes the shape of either solitude, anonymity, isolation, reserve or intimacy) and the beneficial functions that it can support (e.g., emotional regulation, stress recovery, enhancing creativity) (Pedersen, 1997, 1999).

Although the relevance of privacy, as a condition that can provide support for certain positive psychological processes, has been confirmed, very little scientific attention has been paid to the specific functions of privacy. The literature on the psychology of privacy focuses more or less explicitly on the defensive behaviors to avoid the violation of privacy (Altman, 1975; Buslig & Burgoon, 2000; Milgram, 1973; Palen & Dourish, 2003). Very little attention has been paid to the noteworthy function of privacy in supporting some of specific processes involved in the empowerment of personal well-being in terms of restoration and personal growth (Newell, 1994, 1995; Pedersen, 1997, 1999; Westin, 1967). Among the limited extant studies on the positive functions of privacy (Cole & Hall, 2010; Newell, 1994, 1998; Pedersen, 1997, 1999), Newell (1994, 1998) proposed a synthetic functional theory of privacy called the Systems Model of Privacy. According to this model, privacy plays a central role in supporting two classes of psychological processes, “system maintenance” and “system development”, to provide the conditions for both threat release and personal growth (Newell, 1994, 1995, 1998).

This work intends to gain a deeper understanding of the person’s experience of the beneficial functions of privacy, considering the complex overlap between physical and digital environment, as the process of privacy unfolds, from its need to the experience of its effects.

## The “How” and “Why” of Privacy: The Functions of Privacy and the Personal Well-Being

Two main traditions of research on the topic of privacy exist in psychology. Some authors have focused on the “how” of privacy, that is, the mechanisms through which a person seeks and obtains it (Altman, 1975, 1977; Buslig & Burgoon, 2000; Klopfer & Rubenstein, 1977; Petronio, 2003). These mechanisms consist of learned behaviors that serve to regulate social interaction and that vary widely across cultures (Altman, 1977). The primary aim of these behaviors is to protect the person from environmental threats. These protective behavioral mechanisms are prerequisites for allowing the subject to reach a condition of privacy (Newell, 1994). Accordingly, we propose that the need for defense from threats precedes and makes possible the achievement of an experience of privacy. Moreover, the need for the system to be protected from threats is activated by the experience of encroachment, even when anticipated.

A different perspective concerns instead the investigation of the “why” of privacy (Newell, 1994, 1995, 1998; Pedersen, 1999; Westin, 1967); thus, its functions for personal well-being, which people actualize when they achieve this state. This approach investigates the benefits of privacy; therefore, the focus is on the reached state of privacy rather than to the steps required to reach it. Integrating the research interests about the “how” and the “why” of privacy, it is possible to identify the main features that characterize this process from the initial step of seeking it to the final step regarding the contemplation of the effects of its achieved state. Some of the features related to this process of seeking-experiencing, such as the antecedent conditions connected to a desire for privacy (Newell, 1994; Pastalan, 1970), the affective set linked to its requirement (Newell, 1994), the urgency through which the subject seeks it (Newell, 1998), the affective state related to its achievement (Francis & Cooper, 1991; Newell, 1994) and the perceived ability to achieve it, have been investigated in previous studies.

Specifically, the proponents of the approach related to the “why” of privacy (Newell, 1994, 1995, 1998; Pedersen, 1999; Westin, 1967) make a point, which appears to be relevant for the purposes of the current work. They argue that privacy has two main functions for human well-being, (a) a *system maintenance* function, which involves cognitive and emotional processes implicated in the maintenance of the *homeostasis* and in a state of release and (b) a *system development* function, which comprises psychological processes, such as self-conscious processes, involved in personal growth (Cole & Hall, 2010; Hammitt, 2012; Newell, 1998; Pedersen, 1999). As a system maintenance function, privacy supports processes to promote and ensure a satisfying level of *homeostasis* (Cannon, 1932), since it is often associated with the experience of recovery from psychophysiological stress (Ulrich et al., 1991), emotion regulation (Izard & Kobak, 1991), self-regulation and release (Korpela, Kyttä, & Hartig, 2002; Vuorinen, 1990). Reframing the discourse on this topic to reflect the approach of positive psychology, it is possible to state that this type of experience may be associated with the hedonic experience (Kahneman, Diener, & Schwarz, 1999) and the related concept of *subjective well-being* (Diener, 2000), which focus on positive affect and the absence of unpleasant experience. As a system development function, privacy provides the condition to exercise and improve self-conscious processes, like self-evaluation, contemplation of one’s thoughts and emotions (Long, 2000; Long & Averill, 2003; Pedersen, 1997, 1999), problem-solving, creative thought (Edney & Buda, 1976; Ittelson, Proshansky, Rivlin, & Winkel, 1974; Newell, 1994), and spirituality (Pedersen, 1999; Suedfeld, 1982). In this regard, for example, Pedersen found that some psychological processes involved in the positive functioning, such as self-discovery and planning, are best supported by specific privacy forms, such as solitude and isolation conditions, rather than other forms, such as either intimacy or anonymity conditions (Pedersen, 1997).

Considering the positive psychology point of view on this topic, the system development function underlies the idea of well-being as a complex long-term-oriented process of self-development and growth. Thus, privacy presents a particular focus on personal growth (Keyes, 2002; Seligman & Csikszentmihalyi, 2000). This approach to well-being is particularly close to the idea of *psychological well-being* (Ryff & Singer, 1998, 2008), which underlines the positive factors that empower individuals to fulfill self-actualization (Ryan & Deci, 2000).

Following this process approach to privacy, the current study aims to focus on two types of function of privacy: the function of protection from threats and the function related to an achieved condition of privacy. The former is a prerequisite for the achievement of a state of privacy. Compared to the latter, it has attracted the most scientific attention so far. The second category comprises the beneficial functions provided by an achieved condition of privacy, such as the system maintenance and the system development (Newell, 1994, 1995, 1998). Despite the limited extant studies on the benefits related to the experience of privacy, scholars agree on emphasizing the relevance of experiencing privacy for the empowerment of well-being (Cole & Hall, 2010; Long & Averill, 2003; Newell, 1994, 1998; Pedersen, 1997, 1999).

## The Role of the Environment in Shaping the Concept of Privacy: Digital Spaces vs. Physical Environments

Following the theoretical framework in which privacy arises from the interaction between the person and the environment, some authors have been interested in investigating the role of the environment in shaping the features of an experience of privacy. Specifically, it is possible to identify two fields of research concerned with this matter.

First, the issue of privacy has been applied to the digital space domain. In the current study, we used the term “digital environment” to indicate technology-based social interactions, such as computer-mediated-communication (CMC), social-network interaction and communication *via* media and new media (Palen & Dourish, 2003; Sommer, 2002; Tu, 2002b). Information technologies have contributed to the creation of new sets of problems; specifically, they have created a ubiquitous environment where sensible constraints are no longer perceived. Hence, social interaction becomes difficult to manage (Bellotti, 1998; Grudin, 2001; Neumann, 1995; Tu, 2002b), since it has been related to multiple changes in boundary regulation practices (Boyle, 2003; Boyle, Neustaedter, & Greenberg, 2009; Palen & Dourish, 2003). Consequently, an increase in sensitivity to the risks of violation of one’s personal information has occurred (Cole et al., 2001; Röcker & Feith, 2009). From this perspective, the issue of privacy is associated with the problem of managing the information shared about oneself (Altman, 1975, 1977; Röcker & Feith, 2009).

A second line of research considers the influence of the *physical environment* in determining the features of an experience of well-being (Cole & Hall, 2010; Hammitt, 1982, 2012; Kaplan, 1995; Korpela, Hartig, Kaiser, & Fuhrer, 2001; Korpela et al., 2002). From this perspective, the concept of privacy is useful to illustrate the kind of experience that people seek in particular contexts (e.g., in the wilderness) (Hammitt, 2012). In his study, Hammitt (2012) showed that *wilderness privacy* operates as a coping strategy to achieve a desired environmental state, as it works as a boundary regulation mechanism, which serves to fit with one’s needs for space and social interaction (Altman, 1975; Proshansky, Ittleson, & Rivlin, 1976) and for experiencing emotional and cognitive release (Westin, 1967) from everyday stressors and fatigue. Indeed, it is easier to identify strategies to take advantage of the beneficial functions of privacy to experience well-being in the physical environment rather than in this ubiquitous environment, since the former provides well-known sensible coordinates in which one can move in order to manage his/her social activities.

Considering the points made in the present discussion, some relevant questions about the function of privacy and the role of the environment in shaping its experience emerge.

First, it may be interesting to examine the processes related to the functions of privacy as well as the experience of its beneficial effects, as pointed out in the literature.

Second, studies on both the digital and the physical environment reflect two main traditions of research on the topic of privacy in psychology. The “how” of privacy is particularly critical for research on the previously mentioned digital environment. The branch of work concerning privacy in the wilderness and the restorative experience in natural environments can be seen as part of “why” line of research. It would be interesting to go beyond the opposition between negative-positive experiences of privacy in relation to digital vs. physical environment through the investigation of the experience of the person-environment interaction in both types of environment.

## Aims of the Study

The primary purpose of the current study was to investigate the experience of privacy, with a focus on its functions for well-being (i.e., the functions of threat defense, of system maintenance, and of system development). Hence, we proposed to collect data on both the spontaneous definition of privacy and the guided description of its daily experience.

Specifically, we intended to (1) investigate the relevance attributed to each of the two categories of function of privacy, such as the function of defense of the system and the function related to the achieved state of privacy (i.e., function of maintenance and function of development).

Hence, we expected that (a) the function of defense of the system would be mentioned more frequently compared to the function related to a state of achieved state of privacy (i.e., system maintenance function and system development function), since the aspects associated with the protection from threats are easier to identify in one’s daily experience of privacy, and the defense processes take priority over the psychological processes related to an achieved state of privacy (Cannon, 1932; Newell, 1994).

Second, we also expected that (b) considering the two functions related to the achieved state of privacy (i.e., the function of maintenance and the function of development), the function of maintenance would be cited more frequently compared to the function of development, as the effects related to the psychological processes involved in the maintenance of the system, (e.g., the effect of recovery from threats and emotional regulation) are easily to be detected compared to those involved in the development (e.g., the result of self-evaluation and contemplation of one’s thoughts), which comprises long-term processes.

Second aim of the current work was to (2) investigate the role of the environment in shaping the experience of privacy.

According to the literature (Boyle, Neustaedter, & Greenberg, 2009; Palen & Dourish, 2003; Tu, 2002b), we expected that (c) the aspects associated with two positive functions related to a state of achieved privacy would be mentioned less frequently in relation to digital rather than physical environments, since in digital spaces, managing the threats of violation of one’s privacy (i.e., function of protection from threats) is of much concern (Boyle, Neustaedter, & Greenberg, 2009; Palen & Dourish, 2003).

## Method

### Participants and Procedure

The sample comprised 180 Italian volunteer males (*n* = 90) and females (*n* = 90) between the ages of 18 and 50 (*M* = 32.26; *SD* = 10.807). The subjects were heterogeneous in terms of education (bachelor’s degree = 44.44%; high school diploma = 48.33%; middle school diploma = 7.22%) and occupation (student = 28.89%; employee = 52.78%; freelance = 18.33%). The participants were recruited via the Internet. All subjects were Italian and currently living in Italy.

The study was conducted as a web-based-survey (Sue & Ritter, 2007), complying with requirements of anonymity and sampling (Gosling, Vazire, Srivastava, & John, 2004; Seligman, Steen, Park, & Peterson, 2005).

### Instruments

We used two instruments to conduct a free exploration and obtain a guided detailed description of the daily experience of privacy.

The first instrument comprised open-ended questions to investigate the sample’s social representation of privacy through their spontaneous definitions of privacy (Foddy, 1993; Reja, Lozar Manfreda, Hlebec, & Vehovar, 2003; Vinten, 1995). Specifically, it comprised three open questions. The first question concerned the meaning associated with privacy. It was phrased as follows: “Think about the meaning that you give to the word ‘privacy’. Then complete the following sentence: To me, privacy means…” The second open question concerned an experience of privacy violation. It was worded as follows: “Think about an episode in your life in which you felt your privacy being violated, then please write about it”. The third open question dealt with the account of a positive experience of privacy. It was worded as follows: “Think about an episode in your life in which you felt a positive experience of privacy, then please write about it.”

The second instrument was a modified version of an ad hoc privacy questionnaire developed by Newell (1998). It comprised two sections. The first section assessed demographic variables, including age, gender, education, and occupation. The second and final section of the instrument included close-ended questions. These questions assessed the main features that characterize the process of seeking privacy. (a) The *antecedent conditions* connected to a desire for privacy were assessed. Past research (Newell, 1994, 1998; Pastalan, 1970) identified the following antecedent conditions: “Social antecedents”, referring to the concerns for other people, such as overcrowding, need for intimacy, and perceived responsibilities to other people; “Physical conditions*”,* referring to physical factors, which, if experienced, may require the achievement of privacy, such as noise and pollution; “Motivational antecedents”, referring to intentions and desires to do or achieve something; and “Organismic factors”, referring to disturbance in the physiological state of the individual that is unrelated to immediate social conditions, such as major injury, aggression, anxiety state, inability to control one’s action, menses. (b) The *affective set* linked to a requirement of privacy was assessed as another feature of privacy seeking (Newell, 1994). The affective set categories used in past research (Korpela 1991; Newell 1998) included sadness, anger, anxiety, tiredness, positive state, creative thinking, desire to focus on one’s thoughts and desire of intimacy related to a previous condition to the achievement of privacy. (c) The *after affective effects* of the achievement of privacy (Francis & Cooper, 1991; Korpela, 1989, 1991; Newell, 1994, 1998) were derived from past research on privacy experience as well as restorative places. Categories include relaxation, emotionality, tiredness and refreshments experienced after the achievement of a state of privacy. (d) The *perceived ability to achieve* privacy and (e) the *urgency* with which the subject seeks privacy (Newell, 1998) are the primary driver of seeking privacy and consider the following options: distress, concentration, need for creative thinking, and need for intimacy. Questions related to the duration of the average experience of privacy, the frequency of the occurrence of both a need for privacy and a need for sharing a privacy experience with others were not included in the current version of the instrument. The original version of the questionnaire (Newell, 1998) was translated into Italian.

## Data Analysis

The present study used a quali-quantitative analysis of the answers to the open questions. To explore the primary features of the sample’s concept of privacy, the textual corpus obtained from the answers was systematically analyzed using the T-lab 7.2 software for content analysis and text mining^i^. The choice of this instrument was justified by the fact that it allows investigating both the inner structure of a semantic map and the process of meaning making (Lancia, 2004). After the text corpus was set up, two kinds of textual analysis were conducted. First, a cluster analysis, respectively called “Thematic Analysis of Elementary Context”, was performed (Guest & McLellan, 2003; Richards & Richards, 1995). This analysis made it possible to identify the principal themes associated with the sample’s social representation of privacy and their localization into a *semantic space.* This semantic field was drawn up by the interception of two axes explaining the thematic variance of the considered corpus (Gambetti & Graffigna, 2010). Specifically, the main isolated clusters consisted of Elementary Contexts (EC). Each EC had the minimum degree of inner variance and the maximum degree of the external one. Second, a co-occurrence analysis, which is called “Word Association Analysis” (WAA), was conducted to obtain a more precise exploration of the semantic association between some of the principal key words (i.e., Lexical Units, LU) to determine their local meaning. The relationships were measured with a semantic association index (Cosine Coefficient, Salton & McGill, 1984).

The main features that characterize the experience of privacy collected through closed-ended questions were analyzed to explore the sample’s daily experience of privacy. Chi-square analysis was applied to compare the data on the system maintenance type of function of privacy with those on the system development function (Newell, 1994, 1998).

## Results

### Free Exploration of the Experience of Privacy

#### Occurrence of Omitted Answers

The first noticeable result that emerged from the free exploration of the semantic dimensions of privacy concerned the percentage of omitted answers to the open questions. A large portion (44%; *n* = 93) of the sample (*N* = 180) did not answer the question asking them to recount a positive experience of privacy (Question 3) while another significant percentage (23%; *n* = 49) did not answer the question asking them to recount an experience of privacy violation (Question 2). Only 4.25% (*n* = 9) omitted answers to the question about the definition of privacy (Question 1).

#### Cluster Analysis (CA)

**Figure 1:**
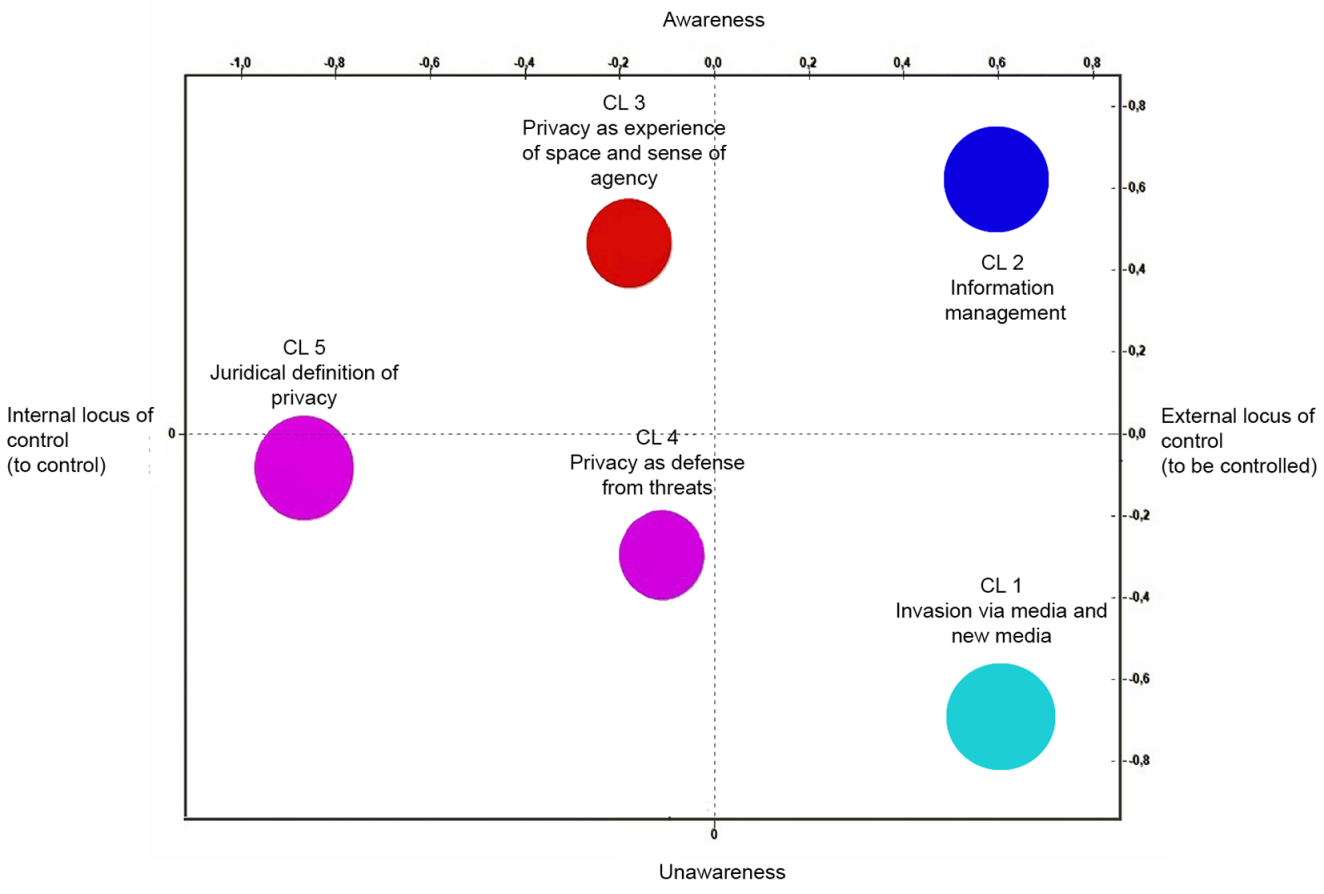
The semantic map of the five clusters obtained with the application of the cluster analysis.

The Thematic Analysis of Elementary Context was carried out on the entire textual corpus, which comprised the answers to the three open-ended questions included in the questionnaire. This analysis allowed for the creation of a semantics map through the individuation of two main factors that were used to organize the amount of textual data (see Figure 1).

- Factor 1 represents the *locus of control over private information* (“*internal locus of control-external locus of control”* axis in Figure 1). Locus of control is *external* if the person perceives himself or herself to play a passive role in the management of his or her private information (right extreme), whereas it is *internal* if the person feels that he or she holds active control over this information (left extreme).
- Factor 2 represents the *level of awareness about one’s own experience* (*“awareness-unawareness*” axis in Figure 1). The bottom of the axis represents a minimum level of awareness (*unawareness*) of several features of one’s daily experience, including the implicit risk associated with the interaction with media and new media and the ability to identify privacy (including its main facet and its thorough definition). Conversely, the top of the axis corresponds to a maximum level of awareness about one’s own experience of privacy.

The mapping of the above criteria resulted in the isolation of five thematic clusters in which the corpus was organized (see Appendix). The first cluster deals with the negative experiences of invasion of privacy via media and new media (e.g., telephone, the Internet, e-mail, and Facebook). These types of experiences increase the perception of violation of privacy due to the subject’s unawareness of the means by which an unknown person can obtain the subject’s personal information (e.g., telephone numbers, personal account information and other identifying data).

The second cluster focuses on the management of private information by others. This cluster includes experiences through which the respondents entitle other persons to handle their own data. The management of private information by third parties can be proper or improper, such as, for example, publishing school results or speaking loudly during a medical examination.

The third, fourth, and fifth clusters deal more directly with the definition of privacy. The third cluster focuses on the experiential and concrete features of privacy. When privacy is achieved, participants have a positive experience; they consider privacy a physical space in which one can exert free will and perceive a sense of agency.

The fourth cluster focuses on a dialectic definition of privacy as a condition of “the lack of privacy violation”. This cluster includes elementary context, which defines privacy as a condition in which “nobody bothers me”, “my secrets are protected”, “nobody violates my personal space”, “nobody can read my personal diary”, and the like.

The fifth cluster centers on the normative aspects associated with privacy. Typical terms in this cluster concern the semantic field of protection and defense against violation of sensitive data. Specifically, the individual protects the data him or herself.

#### Word Association Analysis (WAA)

A Word Association Analysis was conducted to specify and support the outcomes obtained from the CA (see Table 1). Specifically, the associations between the concept of privacy and other concepts related to the entire corpus were studied and measured using a Cosine Coefficient (CC, Salton & McGill, 1984). Considering the four most significant associations with the concept of privacy (“life”, “violation”, “personal”, and “respect”), the results showed that privacy can be represented through three primary semantic dimensions. First, the sample seemed to acknowledge that privacy concerns the most intimate aspects of life and is linked to their daily experience (‘privacy’ and ‘life’, CC = 0.479; ‘privacy’ and ‘personal’, CC = 0.434). Second, privacy was an acknowledged domain; thus, others have to respect it (‘privacy’ and ‘respect’ CC = 0.403). The dimension of respect entails interaction with other people and seems to emphasize the normative aspect of privacy as a juridical right. It also comprises the “need for control” that emerged from the sample’s accounts. The third dimension concerns the violation of a state of privacy, as indicated by the high value of the association between privacy and violation (CC = 0.47). This value suggests that the experience of violation of privacy is an important factor in the definition of the concept. Therefore, the considered association justifies the hypothesis that the concept of violation is often activated when the respondents think about privacy.

**Table 1:** Word Association Analysis (WAA) of the Textual Corpus

Lemma	Translated Lemma	Cosene Coefficient	EC “Privacy”	EC “Privacy” + other lemma
vita	life	0.479	53	42
violazione	violation	0.470	45	38
personale	personal	0.434	103	53
rispettato	respected	0.403	55	36
dati	data	0.388	53	34
informazione	information	0.364	38	27

### Guided Exploration of the Experience of Privacy

Considering the conditions that stimulate the subject to achieve privacy (“antecedent factors to privacy*”*), most participants (*N* = 180) indicated “social factors*”* as the first (43.89%) and “motivational factors*”* as the second (27.22%) reason to pursue privacy, followed by “physical factors*”* (11.67%) and “organismic factors*”* (10.00%).

The “urgency” factor concerns a set of aspects that explain the subjective need to achieve privacy. A large portion (41.11%) of the total sample (*N* = 180) indicated “concentration*”* as the main reason and “stress*”* as the second reason (32.78%) to seek privacy.

Furthermore, the findings suggested that privacy is mostly sought for reasons concerned with “system development*”* (total development = 58.89%) rather than “system maintenance*”* (total maintenance = 40.56%) (Table 2). These reasons differed significantly, as indicated by a chi square of 6.084 with 1df and a p-value of 0.014.

**Table 2:** Urgency to Achieve Privacy, as Identified by the Total Sample (N = 180)

Urgency	Percentage of the total sample (*N* = 180)
System maintenance	
Distress	32.78%
Emotional	7.78%
Total	40.56%
System development	
Concentration	41.11%
Creativity	17.78%
Total	58.89%
**No answer**	0.56%

#### Affect Associated With Antecedents

“Affect associated with the antecedents” refers to the affective experiences of the subject after he/she identifies a need for privacy. According to past research, initial affect associated with a desire for privacy can be both negative (i.e., whether I am experiencing an overall negative affect while I am searching for privacy) or positive (i.e., whether I am experiencing an overall positive affect while I am searching for privacy). Negative affect involves the experiences characterized by a need for restoration, physically, emotionally, and cognitively, while positive affect involves a state characterized by a need for being productive and involved in something important in one’s life. Of the total sample, 36.67% (*N* = 180) indicated the “desire to focus on my thoughts” as the primary reason to seek privacy while 23.33% indicated “tiredness*”* (i.e., “I most often require a period of privacy when I am tired”). Overall, both negative and positive affects associated with antecedents showed equal distribution of the data (Table 3).

#### After Effects Associated With Obtaining the Condition of Privacy

Most participants (75.00%; *N* = 180) evaluated the affective set associated with a condition of achieving privacy positively; conversely, only a small percentage of the sample (22.22%) stated that they felt worse after achieving a condition of privacy. These responses differed significantly, as indicated by a chi square of 51.571 with 1 df and a p-value of 0.001.

“Ability to achieve privacy” refers to the subjects’ assessment of their perceived ability to reach a condition of privacy when needed. Of the total sample (*N* = 180), 85.56% affirmed that they were able to pursue privacy “frequently” and 60% indicated they were able to pursue privacy only “occasionally”. Smaller portions of the sample indicated that they could achieve privacy “rarely” (30%) or “always” (18.89%). Only 4.44% said that they were “never” able to achieve privacy.

**Table 3:** Affective Set Associated to Antecedents, as Identified by the Total Sample (N = 180)

Affective set	Percentage of the total sample (*N* = 180)
Negative Affect	
Sad	9.44%
Angry	10.00%
Anxious	4.44%
Tired	23.33%
Total	47.22%
Positive Affect	
Positive	2.78%
Creative	6.11%
Focused	36.67%
Intimate	2.22%
Total	47.78%
**No answer**	5.00%

**Table 4:** After Effect Associated to a Condition of Obtained Privacy, as Identified by the Total Sample (N = 180)

After effect associated to an obtained condition of privacy	Percentage of the total sample (*N* = 180)
Positive	
More relaxed	45.56%
Less emotional	0.56%
Refreshed	12.22%
Back to normal	16.67%
Total	75.00%
Negative	
Less relaxed	0.56%
More emotional	10.56%
More tired	0.00%
The same as before	11.11%
Total	22.22%
**No answer**	2.78%

## Discussion

The present study aimed to explore the representation of privacy in an Italian sample and to investigate the importance of the two categories of functions of privacy (i.e., function of protection from threats, functions related to an achieved state of privacy) while considering the results of the spontaneous definition of privacy and those of the guided description of a daily experience of privacy. Specifically, participants were expected to make a positive use of privacy in their daily experience (Cole & Hall, 2010; Newell, 1998).

According to the first aim of the current study, we wanted to explore the relevance of each of the two types of positive functions of privacy (i.e., the function of defense from threats and the functions related to the achieved state of privacy, such as the “system maintenance” function and the “system development” function) based on the participants’ responses collected via open questions and pre-coded questions.

Overall, the findings that arose from the sample’s spontaneous definitions showed that the subjects seem to attribute most relevance to the function of protection from threats compared to the two functions related to an achieved state of privacy. Specifically, the subjects’ representations of privacy contained primarily references to both the defense from threats and the protection of personal information (Figure 1, Table 1). Figure 1 shows that four of the five clusters generated by the clusters analysis conducted in the current study, which are the first, the second, the forth, and the fifth cluster, dealt more or less explicitly with the aspects related to the defense against threats/protection of personal information (e.g., the fourth cluster collects strategies pertaining to protection, such as the behaviors of protecting the access to one’s personal diary or to one’s room). Similarly, the findings from the word association analysis (Table 1) showed the semantic closeness between the aspects concerning the protection of the most intimate aspects of life (e.g., personal data, intimate relationships information) and the concept of privacy. The sample’s representations of privacy were built on its normative features, such as the management of personal information and the defensive mechanisms to protect one’s intimacy from threats (e.g., “Privacy occurs when my personal space boundaries are respected”, “I usually protect my personal information putting passwords everywhere”), as it is shown in the findings that arose from the content analysis (Appendix and Table 1). The conceptualization of privacy as a process aimed at protecting the most private information and defending the system from social threats, which arose from the participants’ spontaneous accounts, reflects a traditional theoretical definition of privacy focused on the mechanisms used to protect the system from social threats (Altman, 1975; Hammitt, 2012). The “protection from social threats” nature of privacy is also shown in the guided exploration of the daily experience of privacy, where the stressful events that concern the interaction with other people (“social antecedents*”*), such as the condition of overcrowding, were most frequently reported as antecedents related to the need for a period of privacy, as indicated by the participants.

Considering the second function of privacy, the findings of the spontaneous definitions refer to the beneficial qualities of privacy related to an achieved state of privacy (i.e., function of maintenance, function of development). This function of privacy has been mentioned less frequently compared to the function of protection from threats (Figure 1, Appendix). These data confirms the first hypothesis (a), since the participants cited both functions of privacy (i.e., the function of protection and the functions related to the achieved state of privacy) in their spontaneous accounts. Moreover, the protection from threats is the most relevant concern that constitutes the great part of the participants’ representation of privacy, and it may guide the most part of their privacy-related behaviors. Participants referred to the function associated with the achieved state of privacy mostly in their descriptions of the activities that one may carry out in private (Figure 1, Cluster 3). Therefore, these findings seem to suggest that satisfying the requirements of the protection of the system from threats (i.e., function of protection) is a prerequisite for experiencing the beneficial qualities provided by a period of privacy (i.e., maintenance of the system, development of the system). This proposed interpretation needs to be investigated in future studies.

Through the second hypothesis, (b) we expected that focusing on the two functions of privacy related to the achieved state of privacy (i.e., the function of maintenance and the function of development), the function of maintenance would be mentioned more frequently compared to the system development function because the aspects associated with the maintenance dimension are easier to identify in one’s daily experience of privacy.

Overall, the data shows that participants assigned an equal importance to both of the positive functions related to the achieved state of privacy, considering the spontaneous definitions as well as the guided exploration of the daily experience. It was found that subjects reported personal experiences related to the system maintenance function of privacy (e.g., “When I experience a period of privacy, then I feel regenerated”, “I usually seek privacy when something stressful occurred and I need to recover”) as well as to the system development function of privacy (e.g., “I feel privacy when I am alone in my own room. Once there, I can contemplate about my thoughts and put things in perspective”, “Cultivate a friendship needs sharing one’s privacy”).

Furthermore, the outcomes of the pre-coded questions provided support for the high relevance ascribed to both beneficial functions of privacy related to an achieved state of privacy (i.e., system maintenance function, system development function). Further support was obtained through the equal distribution of the answers across negative and positive affects associated with the antecedent conditions of privacy (Table 3). In particular, we speculated that if a negative affective state, such as anxiety or anger, is experienced along with a need for privacy, then it is more likely that privacy would be required for its “systems maintenance functions” (e.g., in order to regulate one’s emotions or to recover from overstimulation). Conversely, if a person feels positive affect, such as interest or curiosity in something, while he or she perceives a need for privacy, he or she would likely require privacy for its “systems development functions” (e.g., in order to commit to a creative task or to find concentration).

The results concerning the beneficial functions of privacy related to the achieved state of privacy can be also explained by the theoretical approach offered by the field of positive psychology (Kashdan, Biwas-Diener, & King, 2009; Kashdan & Steger, 2007). According to the subjective well-being perspective of positive psychology (Diener, 2000; Kahneman, Diener, & Schwarz, 1999), we found that an achieved period of privacy provides a positive experience, since it offers the opportunity for cognitive, emotional, and physical regeneration (Table 4). Specifically, considering the spontaneous descriptions of the experience of privacy, subjects reported that they perceive privacy when they “feel in control over the environment”, “perceive safety”, as well as experience positive states, such as satisfaction after a reached period of privacy. This data is in line with previous findings (i.e., the concept of “therapeutic value to privacy”, Newell, 1998, p. 367).

Moreover, the findings are also consistent with the psychological well-being perspective of positive psychology (Ryff & Singer, 1998, 2008), since individuals seek privacy while looking for psychological conditions that lead to self-actualization (Waterman, 1993) and personal growth (Keyes, 2002), such as self-monitoring, self-contemplation (Long, 2000; Long & Averill, 2003; Pedersen, 1997, 1999), and problem solving (Newell, 1994, 1998). According to the abovementioned explanation, the primary motive to seek privacy is related to personal growth (i.e., systems development function of privacy), as the subjects indicated that privacy is sought most frequently to find creative solutions to problems and achieve some important life purposes (Table 2).

In addition, another unexpected result concerns the relevance of the two beneficial functions of privacy to the achieved state of privacy, as the participants mentioned the aspects related to personal growth (i.e., system development) more frequently than they did aspects related to maintenance of the system (i.e., system maintenance) [Chi square (df = 1) = 6.084; p = 0.01] (Table 2).

Overall, these findings disconfirmed the second hypothesis (b), which proposed that the system maintenance function of privacy would be mentioned more frequently compared to the system development function, supporting previous findings (Newell, 1994). Thus, the results confirmed that privacy is equally important to the Italian sample, both for its maintenance function and for its system development it can support, even if it is more frequently required to accomplish personal growth processes, as indicated by the sample’s daily experiences.

According to the second aim (2) regarding the experience of the positive functions of privacy related to the type of environment (digital vs. physical environment), privacy seems to be most frequently violated in the digital environment where the negative consequences are intensified by the perception of low control over who is managing one’s information and by the way in which personal information is managed according with the structural features of the environment. In particular, the interactions provided by the new media seem to facilitate the intrusion into the private space and to enhance the perception of the loss of control (Figure 1). Considering these results, it may be possible to state that subjects viewed the function of one’s protection against threats as a defense process that is highly related to this type of environment. Conversely, references to the beneficial qualities of privacy, reflecting systems development function and systems maintenance function, were related to the physical environment, as reported by the Italian sample (Figure 1). In particular, the material features of the environment, such as the physical space that provides physical barriers that are easy to use to exercise control over the access to one’s space and information (e.g., “[…] when I spend time alone in my room and I can decide who to let in”), were mostly associated with positive experiences. The benefits provided by a state of privacy achieved are easily recognized (Figure 1). These findings confirm the third hypothesis of the current study and support the existing literature on the topic (Cole & Hall, 2010; Hammitt, 2012; Palen & Dourish, 2003; Tu, 2002b) that identified the function of privacy related to system protection from threats in digital context and reported the benefits of a privacy experience in the physical environments.

Moreover, the data is in line with the abovementioned idea that participants seem to be mostly concerned with the control over the access to their personal information, and they recognize the beneficial qualities of privacy while they experience it. However, when it is difficult to exercise control due to the environmental features of the setting of the interaction, such as in digital environments (vs. physical environments), they focus on the strategies to assure their protection from threats rather than to identify benefits of privacy.

These findings are also consistent with the idea that the function of protection of privacy is a precondition for achieving privacy and thus its beneficial qualities for well-being.

Finally, the role of the environment in shaping the experience of privacy provides the possibility to confirm the conception of privacy as a socio-environmental construct, which depends on both personal and environmental factors as well as on their interaction (Pedersen 1997, 1999).

## Conclusion

The present study considers privacy as a supportive condition for some psychological processes involved in human well-being, such as increasing the sense of control over the environment (Altman, 1975; Proshansky, Ittleson, & Rivlin 1976), self-regulation (Korpela, Kyttä, & Hartig, 2002) and enhancing creativity (Edney & Buda, 1976; Ittelson, Proshansky, Rivlin, & Winkel, 1974). The results support previous studies conducted on the role of privacy in individuals’ well-being (Altman, 1975; Edney & Buda, 1976; Hammitt, 2012; Newell, 1994, 1995, 1998; Pedersen, 1997; Westin, 1967).

This study has some limitations. First shortcoming is related to the self-reported method we chose in order to satisfy the purposes of the study, such as the exploration of the benefits of privacy in the subjects’ daily experience. Indeed, it would be useful to integrate the obtained data with more objective types of measures, such as observational measures or cognitive measures of performance in specific psychological processes (e.g., creative thinking), to estimate the enhancement of these processes after an achieved period of privacy.

A further problem is related to the instrument that we used to collect the data about the guided exploration of the subjects’ daily experience of privacy. We considered an instrument taken from past research (Newell, 1998) as the basis of our investigation. Two main problems related to the use of this instrument need to be considered. First, we did not investigate the defensive function of privacy. This choice was justified by the fact that the defensive function of privacy has been broadly investigated in the past. Thus, it would be more interesting to investigate the second type of function of privacy, such as that concerned with the benefits related to an achieved state of privacy, since it has attracted only little interest so far. Nevertheless, the present work could be expanded by including questions about the defensive function of privacy. Second, some suggestions for the improvement of the answer categories used in the instrument arose from the data collected though the free exploration of the daily experience of privacy. For instance, the answer categories could incorporate the effects related to an experienced state of privacy. This information could be integrated in the next version of the pre-coded instrument.

However, the current work contributes to the existing literature on the psychological experience of privacy.

First, the study used the quali-quantitative analysis, which proved to be helpful for investigating more deeply about the social representation of privacy and the use of privacy in daily life. It provides data that offer a thorough and comprehensive overview of the subjects’ experience of privacy. Thus, the findings from this work could be used to inform further studies on the functions and process of privacy. For instance, we propose a theory of the process of privacy based on its effects on well-being. It would be interesting to verify the proposed theory in future research.

Second, the study focused on the unusual facets of the construct of privacy. Privacy can be considered a double psychological concept that has received much attention largely because of its protective function (Altman, 1977; Boyle, Neustaedter, & Greenberg, 2009; Palen & Dourish, 2003; Röcker & Feith, 2009) while its unusual side related to the benefits of an experienced state of privacy has been neglected (Newell, 1994). We believe that the beneficial features of privacy are worthy of studying, as they can have remarkable practical implications, for instance, by increasing the attention to conditions facilitating a person-environment interaction that would support privacy in critical contexts, such as schools, workplaces, and health-care environments. We also believe that positive psychology can offer the theoretical and practical instrument to investigate privacy as a positive experience.

In conclusion, some practical indications arise from the current work. Overall, our findings seem to suggest that people are mostly concerned with the protection of privacy (i.e., function of defense of the system) rather than with well-being related to an experience of privacy (i.e., function of maintenance of the system, function of development of the system). Thus, future research should investigate the ways in which the control over the social interaction could be assured, especially in specific kind of environments where it might be difficult to manage access to one’s personal information, such as the digital environments, in order to enhance the benefits offered by an achieved condition of privacy.

## Appendix

### Lemmas Featuring in the Five Conceptual Clusters Obtained With the Application of the Cluster Analysis

**Table A1:** Cluster 1 collects the sample’s experience of invasion of privacy via media and new media

Lemma and Variable	Translated lemma and variable	Chi^2^	EC in cluster	EC in total
e-mail	e-mail	49.827	18	21
ricevere	receive	40.992	13	14
domanda 2	question 2	34.024	55	128
collega	colleague	32.687	14	18
chiamare	call	26.299	10	12
facebook	facebook	23.439	8	9
internet	internet	22.849	10	13
sito	website	22.849	10	13
amico	friend	13.138	13	25
accorgersi	notice	13.122	5	6
aprire	open	13.122	5	6

**Table A2:** Cluster 2 emphasizes the management of private information by other people, who can be entailed to manage one owns data or not

Lemma	Translated lemma	Chi^2^	EC in cluster	EC in total
casa	home	37.811	21	31
esame	exam	25.994	11	14
telefonata	call	23.541	23	44
ospedale	hospital	19.884	7	8
continuo	continue	17.617	5	5
camera	room	17.346	10	15
nome	name	17.346	10	15
cognome	surname	16.455	6	7
numero	number	15.207	10	16
contattare	contact	11.762	8	13
matricola	registration number	9.891	5	7
esiti	result	9.729	4	5
università	university	9.729	4	5

**Table A3:** Cluster 3 concerns the experience of an achieved state of privacy, which is characterized by a sense of agency in managing the boundaries of its own personal space

Lemma	Translated lemma	Chi^2^	EC in Cluster	EC in total
vivere	live	49.875	11	12
spazio	space	38.647	21	40
tempo	time	25.235	13	24
dedicare	spend	20.452	4	4
piacere	like	14.804	4	5
ritagliare	consecrate	14.804	4	5
invadere	invade	13.943	6	10
sicurezza	safety	13.943	6	10
momento	while	13.892	13	32
noi	we	12.352	12	30

**Table A4:** Cluster 4 collects the sample’s experience of privacy as a condition of “lack of violation of privacy”

Lemma and variable	Translated lemma and variable	Chi^2^	EC in cluster	EC in total
segreto	secret	41.725	9	11
social network	social network	23.748	5	6
leggere	read	17.923	4	5
diario	diary	17.415	6	10
episodio	episode	15.481	5	8
domanda 3	question 3	11.843	20	71
confidenza	intimacy	8.693	3	5
trattamento	deal	7.538	5	12
divulgare	tell	7.250	6	16

**Table A5:** Cluster 5 focuses on the juridical features of privacy with a specific emphasis on the act of protecting sensitive data from threats

Lemma and Variable	Translated lemma and variable	Chi^2^	EC in cluster	EC in total
sfera	(private) domain	55.399	23	26
domanda 1	question 1	52.104	50	85
rispettare	respect	39.072	43	77
informazione	information	30.762	27	44
personale	personal	28.688	64	145
individuo	subject	28.092	11	12
diritto	right	24.318	11	13
dato sensibile	sensitive data	23.880	12	15
riservatezza	discretion	19.555	15	23
scegliere	choose	19.322	8	9
decisione	decision	18.825	13	19
libertà	freedom	16.407	12	18
protezione	protection	15.892	8	10
tutela	defense	14.056	11	17

## References

[r1] Altman, I. (1975). *The environment and social behavior.* Monterey, CA, USA: Brooks/Cole.

[r2] Altman I. (1977). Privacy regulation: Culturally universal or culturally specific? Journal of Social Issues, 33, 66–84. doi:. 10.1111/j.1540-4560.1977.tb01883.x

[r3] Bellotti, V. (1998). Design for privacy in multimedia computing and communications environments. In P. Agre & M. Rotenberg (Eds.), *Technology and privacy: The new landscape* (pp. 63-93). Cambridge, MA, USA: MIT Press.

[r4] Boyle, M. (2003). *A shared vocabulary for privacy*. Paper presented at the Workshop on Ubicomp Communities: Privacy as Boundary Negotiation. 5th International Conference on Ubiquitous Computing, Seattle, WA, USA.

[r5] Boyle, M., Neustaedter, C., & Greenberg, S. (2009). Privacy factors in video-based media spaces. In S. Harrison (Ed.), *Media space: 20+ years of mediated life* (pp. 97-122). 10.1007/978-1-84882-483-6_7

[r6] Buslig, A. L., & Burgoon, J. K. (2000). Aggressiveness in privacy-seeking behavior. In S. Petronio (Ed.), *Balancing the secrets of private disclosures* (pp. 181-196). Hillsdale, NJ, USA: Lawrence Erlbaum.

[r7] Cannon, W. B. (1932). *The wisdom of the body.* London, United Kingdom: Kegan Paul.

[r8] Cole D. N. Hall T. E. (2010). Privacy functions and wilderness recreation: Use density and length of stay effects on experience. Ecopsychology, 2(2), 67–75. doi:. 10.1089/eco.2010.0003

[r9] Cole, J. I., Suman, M., Schramm, P., Lunn, L., Coget, J.-F., Firth, D., . . . Aquino, J.-S. (2001). *The UCLA Internet report: Surveying the digital future: Year two*. Los Angeles, CA, USA: UCLA Center for Communication Policy.

[r10] Demirbas O. O. Demirkan H. (2000). Privacy dimensions: A case study in the interior architecture design studio. Journal of Environmental Psychology, 20, 53–64. doi:. 10.1006/jevp.1999.0148

[r11] Diener E. (2000). Subjective well-being. American Psychologist, 55, 34–43. doi:. 10.1037/0003-066X.55.1.34 11392863

[r12] Edney J. J. Buda M. A. (1976). Distinguishing territoriality and privacy: Two studies. Human Ecology, 4, 283–296. doi:. 10.1007/BF01557915

[r13] Foddy, W. (1993). *Constructing questions for interviews and questionnaires: Theory and practice in social research*. Cambridge, United Kingdom: Cambridge University Press.

[r14] Francis, C., & Cooper, C. (1991). Places people take their problems. In J. Urbina Soria, P. Ortega-Andeane, & R. Bechtel (Eds.), *Proceedings of the Twenty-Second Annual Conference of the Environmental Design Research Association* (pp. 178-184). Oaxtepec, Mexico: Environmental Design Research Association.

[r15] Gambetti R. C. Graffigna G. (2010). The concept of engagement: A systematic analysis of the ongoing marketing debate. International Journal of Market Research, 52(6), 801–826. doi:. 10.2501/S147078531020166

[r16] Glaser, B. J., & Strauss, A. L. (1967). *The discovery of grounded theory: Strategies for qualitative research.* New York, NY, USA: Aldine de Gruyter.

[r17] Gosling S. D. Vazire S. Srivastava S. John O. P. (2004). Should we trust Web-based studies? A comparative analysis of six preconceptions about Internet questionnaires. The American Psychologist, 59, 93–104. doi:. 10.1037/0003-066X.59.2.93 14992636

[r18] Grudin J. (2001). Desituating action: Digital representation of context. Human-Computer Interaction, 16, 269–286. doi:. 10.1207/S15327051HCI16234_10

[r19] Guest G. McLellan E. (2003). Distinguishing the trees from the forest: Applying cluster analysis to thematic qualitative data. Field Methods, 15(2), 186–201. doi:. 10.1177/1525822X03015002005

[r20] Hammitt W. E. (1982). Cognitive dimensions of wilderness solitude. Environment and Behavior, 14, 478–493. doi:. 10.1177/0013916582144005

[r21] Hammitt, W. E. (2012). Naturalness, privacy and restorative experiences in wilderness: An integrative model. In D. N. Cole (Eds.), *Wilderness visitor experiences: Progress in research and management* (pp. 62-69). Fort Collins, CO, USA: U.S. Department of Agriculture, Forest Service, Rocky Mountain Research Station.

[r22] Ittelson, W. H., Proshansky, H. M., Rivlin, L. G., & Winkel, G. H. (1974). *An introduction to environmental psychology*. New York, NY, USA: Holt, Rinehart & Winston.

[r23] Izard, C. E., & Kobak, R. R. (1991). Emotions system functioning and emotion regulation. In J. Garber & K. A. Dodge (Eds.), *The development of emotion regulation and dysregulation* (pp. 303-321). New York, NY, USA: Cambridge University Press.

[r24] Johnson, C. A. (1974). *Privacy as personal control*. Paper presented at the Environmental Design Research Association, Milwaukee, WI, USA.

[r25] Kahneman, D., Diener, E., & Schwarz, N. (1999). *Well-being: The foundations of hedonic psychology.* New York, NY, USA: Russel Sage Foundation.

[r26] Kaplan S. (1995). The restorative benefits of nature: Toward an integrative framework. Journal of Environmental Psychology, 15, 169–182. doi:. 10.1016/0272-4944(95)90001-2

[r27] Kashdan T. B. Biwas-Diener R. King L. A. (2009). Reconsidering happiness: The costs of distinguishing between hedonics and eudaimonia. The Journal of Positive Psychology, 3(4), 219–233. doi:. 10.1080/17439760802303044

[r28] Kashdan T. B. Steger M. F. (2007). Curiosity and pathways to well-being and meaning in life: Traits, states, and everyday behaviors. Motivation and Emotion, 31, 159–173. doi:. 10.1007/s11031-007-9068-7

[r29] Keyes, C. L. M. (2002). Complete mental health: An agenda for the 21st century. In C. L. M. Keyes & J. Haidt (Eds.), *Flourishing: Positive psychology and the live well-lived* (pp. 293-312). Washington, DC, USA: American Psychological Association.

[r30] Klopfer P. H. K. Rubenstein D. I. (1977). The concept of privacy and its biological basis. Journal of Social Issues, 33, 52–65. doi:. 10.1111/j.1540-4560.1977.tb01882.x

[r31] Korpela K. M. (1989). Place-identity as a product of environmental self-regulation. Journal of Environmental Psychology, 9, 241–256. doi:. 10.1016/S0272-4944(89)80038-6

[r32] Korpela, K. M. (1991). Are favorite places restorative environments? In J. Urbina-Soria, P. Ortega-Andeane, & R. Bechtel (Eds.), *Healthy environments: Proceedings of the 22nd annual conference of the Environmental Design Research Association* (pp. 371-377). Oklahoma City, OK, USA: EDRA.

[r33] Korpela K. M. Hartig T. Kaiser F. G. Fuhrer U. (2001). Restorative experience and self-regulation in favorite places. Environment and Behavior, 33, 572–589. doi:. 10.1177/00139160121973133

[r34] Korpela K. M. Kyttä M. Hartig T. (2002). Restorative experience, self-regulation, and children’s place preferences. Journal of Environmental Psychology, 22, 387–398. doi:. 10.1006/jevp.2002.0277

[r35] Lancia, F. (2004). *Strumenti per l’analisi dei testi: Introduzione all’uso di T-LAB* [Tools for text analysis: An introduction to T-Lab]. Milano, Italy: Franco Angeli.

[r36] Long, C. R. (2000). *A comparison of positive and negative episodes of solitude* (Unpublished master’s thesis). University of Massachusetts, Amherst, MA, USA.

[r37] Long C. R. Averill J. R. (2003). Solitude: An explanation of benefits of being alone. Journal for the Theory of Social Behaviour, 33(1), 21–44. doi:. 10.1111/1468-5914.00204

[r38] Milgram, S. (1973). Introduction to Chapter 2. In W. H. Ittelson (Ed.), *Environment and cognition*. New York, NY, USA: Seminar Press.

[r39] Neumann, P. (1995). *Computer-related risks.* New York, NY, USA: ACM Press.

[r40] Newell P. B. (1994). A systems model of privacy. Journal of Environmental Psychology, 14, 65–78. doi:. 10.1016/S0272-4944(05)80199-9

[r41] Newell P. B. (1995). Perspectives on privacy. Journal of Environmental Psychology, 15, 87–104. doi:. 10.1016/0272-4944(95)90018-7

[r42] Newell P. B. (1998). A cross-cultural comparison of privacy definitions and functions: A systems approach. Journal of Environmental Psychology, 18, 357–371. doi:. 10.1006/jevp.1998.0103

[r43] Palen, L., & Dourish, P. (2003). Unpacking privacy for a networked world. In *Proceedings of the Conference on Human Factor in Computing Systems (CHI 2003, Ft Lauderdale)* (pp. 129-137). New York, NY, USA: ACM Press.

[r44] Pastalan, L. A. (1970). Privacy as an expression of human territoriality. In L. A. Pastalan & D. H. Carson (Eds.), *Spatial behavior of older people* (pp. 88-101). Ann Arbor, MI, USA: University of Michigan Press.

[r45] Pedersen D. M. (1997). Psychological functions of privacy. Journal of Environmental Psychology, 17(2), 147–156. doi:. 10.1006/jevp.1997.0049

[r46] Pedersen D. M. (1999). Model for types of privacy by privacy functions. Journal of Environmental Psychology, 19(4), 397–405. doi:. 10.1006/jevp.1999.0140

[r47] Petronio, S. (2003). *Boundaries of privacy: Dialectics of disclosure.* Albany, NY, USA: State University of New York Press.

[r48] Proshansky, H. M., Ittleson, W. H., & Rivlin, L. G. (1976). Freedom of choice and behavior in a physical setting. In H. M. Proshansky, W. H. Ittleson, & L. G. Rivlin (Eds.), *Environmental psychology: People and their physical setting* (pp. 170-181). New York, NY, USA: Holt, Rinehart, & Winston.

[r49] Reja, U., Lozar Manfreda, K., Hlebec, V., & Vehovar, V. (2003). Open-ended vs. closed-ended questions in Web questionnaires. In A. Ferligoj & A. Mrvar (Eds.), *Developments in applied statistics* (pp. 159-177). Ljubljana, Slovenia: FDV.

[r50] Richards, T., & Richards, L. (1995). Using hierarchical categories in qualitative data analysis. In U. Kelle (Ed.), *Computer-aided qualitative data analysis: Theory, methods, and practice* (pp. 80-95). Thousand Oaks, CA, USA: Sage.

[r51] Röcker, C., & Feith, A. (2009). Revisiting privacy in smart spaces: Social and architectural aspects of privacy in technology-enhanced environments. In P. S. Sandhu (Ed.), *Proceedings of the International Symposium on Computing, Communication and Control (ISCCC ‘09), Singapore, October 9-11, 2009* (pp. 201-205). Singapore, Singapore: International Association of Computer Science and Information Technology.

[r52] Ryan R. M. Deci E. L. (2000). The darker and brighter sides of human existence: Basic psychological needs as a unifying concept. Psychological Inquiry, 11(4), 319–338. doi:. 10.1207/S15327965PLI1104_03

[r53] Ryff C. D. Singer B. H. (1998). The contours of positive human health. Psychological Inquiry, 9, 1–28. doi:. 10.1207/s15327965pli0901_1

[r54] Ryff C. D. Singer B. H. (2008). Know thyself and become what you are: A eudaimonic approach to psychological well-being. Journal of Happiness Studies, 9, 13–39. doi:. 10.1007/s10902-006-9019-0

[r55] Salton, G., & McGill, M. J. (1984). *Introduction to modern information retrieval*. New York, NY, USA: McGraw-Hill.

[r56] Seligman M. E. P. Csikszentmihalyi M. (2000). Positive psychology: An introduction. American Psychologist, 55, 5–14. 10.1037/0003-066X.55.1.5 11392865

[r57] Seligman M. E. P. Steen T. A. Park N. Peterson C. (2005). Positive psychology progress: Empirical validation of interventions. American Psychologist, 60(5), 410–421. doi:. 10.1037/0003-066X.60.5.410 16045394

[r58] Sommer, R. (2002). Personal space in a digital age. In R. B. Bechtel & A. Churchman (Eds.), *Handbook of environmental psychology* (pp. 647-660). New York, NY, USA: John Wiley & Sons.

[r59] Sue, V. M., & Ritter, L. A. (2007). *Conducting online surveys*. Los Angeles, CA, USA: Sage.

[r60] Suedfeld, P. (1982). Aloneness as a healing experience. In L. A. Peplau & D. Perlman (Eds.), *Loneliness: A sourcebook of current theory, research, and therapy* (pp. 54-67). New York, NY, USA: Wiley and Sons.

[r61] Tu C. H. (2002 b). The measurement of social presence in an online learning environment. International Journal on E-Learning, 1(2), 34–45.

[r62] Ulrich R. S. Simons R. F. Losito B. D. Fiorito E. Miles M. A. Zelson M. (1991). Stress recovery during exposure to natural and urban environments. Journal of Environmental Psychology, 11, 201–230. doi:. 10.1016/S0272-4944(05)80184-7

[r63] Vinten G. (1995). Open versus closed questions – An open issue? Management Decision, 33(4), 27–31. doi:. 10.1108/00251749510084653

[r64] Vuorinen, R. (1990). *Persoonallisuus ja minuus* [Personality and self]. Juva, Finland: WSOY.

[r65] Waterman A. S. (1993). Two conceptions of happiness: Contrasts of personal expressiveness (eudaimonia) and hedonic enjoyment. Journal of Personality and Social Psychology, 64, 678–691. doi:. 10.1037/0022-3514.64.4.678

[r66] Westin, A. (1967). *Privacy and freedom*. New York, NY, USA: Atheneum.

